# Shankopathies in the Developing Brain in Autism Spectrum Disorders

**DOI:** 10.3389/fnins.2021.775431

**Published:** 2021-12-22

**Authors:** Yukti Vyas, Juliette E. Cheyne, Kevin Lee, Yewon Jung, Pang Ying Cheung, Johanna M. Montgomery

**Affiliations:** ^1^Department of Physiology, Faculty of Medical and Health Sciences, Centre for Brain Research, University of Auckland, Auckland, New Zealand; ^2^Department of Pharmacology, Faculty of Medical and Health Sciences, University of Auckland, Auckland, New Zealand

**Keywords:** synapse, autism (ASD), shank, development, glutamate

## Abstract

The SHANK family of proteins play critical structural and functional roles in the postsynaptic density (PSD) at excitatory glutamatergic synapses. Through their multidomain structure they form a structural platform across the PSD for protein–protein interactions, as well as recruiting protein complexes to strengthen excitatory synaptic transmission. Mutations in *SHANKs* reflect their importance to synapse development and plasticity. This is evident in autism spectrum disorder (ASD), a neurodevelopmental disorder resulting in behavioural changes including repetitive behaviours, lack of sociability, sensory issues, learning, and language impairments. Human genetic studies have revealed ASD mutations commonly occur in *SHANKs*. Rodent models expressing these mutations display ASD behavioural impairments, and a subset of these deficits are rescued by reintroduction of *Shank* in adult animals, suggesting that lack of SHANK during key developmental periods can lead to permanent changes in the brain’s wiring. Here we explore the differences in synaptic function and plasticity from development onward in rodent *Shank* ASD models. To date the most explored brain regions, relate to the behavioural changes observed, e.g., the striatum, hippocampus, sensory, and prefrontal cortex. In addition, less-studied regions including the hypothalamus, cerebellum, and peripheral nervous system are also affected. Synaptic phenotypes include weakened but also strengthened synaptic function, with NMDA receptors commonly affected, as well as changes in the balance of excitation and inhibition especially in cortical brain circuits. The effects of shankopathies in activity-dependent brain wiring is an important target for therapeutic intervention. We therefore highlight areas of research consensus and identify remaining questions and challenges.

## Introduction

Autism spectrum disorders (ASDs) are characterised by deficits in social communication and interactions, as well as repetitive and restrictive behaviours (American Psychiatric Association, 2013). The prevalence of ASD is 1 in 54 (Maenner et al., 2020). When genetic mutations implicated in ASD are functionally grouped, many converge on specific biological pathways involved in neuronal connectivity and synaptic plasticity, thus leading to the synaptic hypothesis of ASD (Bourgeron, 2009). The SRC homology 3 (SH3) and multiple Ankyrin repeat domain (SHANK) family of proteins are known as organisers of excitatory glutamatergic synapses (Boeckers et al., 1999a,b; Naisbitt et al., 1999). There are three known *SHANK* isoforms: *SHANK1* (*ProSAP3*; chromosome 19q13.33), *SHANK2* (*ProSAP1*; c.11q13.3), and *SHANK3* (*ProSAP2*; c.22q13.3). However, multiple intragenic promoters and alternate splicing lead to an extensive array of SHANK protein isoforms (Lim et al., 1999; Sheng and Kim, 2000; Jiang and Ehlers, 2013; Wang et al., 2014), that show specific protein interactions (Boeckers et al., 2005; Shi et al., 2017). Structurally, SHANK proteins have 63–87% amino acid sequence homology and a characteristic domain sequence including the SHANK/ProSAP N-terminal domain, multiple Ankyrin (ANK) repeats, SH3 domain, PDZ domain, a proline-rich region, and a sterile alpha motile (SAM) domain (Sheng and Kim, 2000; Mameza et al., 2013). Through these protein binding domains, SHANKS interact with receptors, ion channels, cytoskeletal proteins, and scaffolding proteins, thereby contributing to the integrity and molecular composition of excitatory glutamatergic synapses (Jiang and Ehlers, 2013). For example, the PDZ domain in SHANKs can bind to the adaptor protein guanylate kinase-associated protein (GKAP; Naisbitt et al., 1999), which in turn, through the interaction with the postsynaptic density molecule 95 (PSD95; Naisbitt et al., 1999), associate with glutamatergic receptors, such as NMDARs (Kornau et al., 1995) and AMPARs (Uemura et al., 2004; for review see Boeckers et al., 2002).

SHANK1 is exclusively expressed in the brain, whereas SHANK2 and SHANK3 are predominantly found in the brain but are also expressed in the kidneys, liver, testis, pancreas, spleen, and heart (Lim et al., 1999; Yao et al., 1999; Zitzer et al., 1999). Within the brain, SHANK1 is highly expressed in the hippocampus, cortex, and amygdala, at moderate levels in the substantia nigra and thalamus, and at very low levels in the striatum, cerebellum and subthalamic nucleus (Zitzer et al., 1999). SHANK2 and SHANK3 are abundantly expressed in the cortex, hippocampus, and olfactory bulb. In the striatum, SHANK3 is most abundant, but there is also SHANK2 expression (Peça et al., 2011; Schmeisser et al., 2012; Monteiro and Feng, 2017). Within the cerebellum, SHANK2 is expressed in Purkinje cells whereas SHANK3 is found only in the granule cell layer (Boeckers et al., 1999a,b; Kleijer et al., 2014).

**FIGURE 1:**
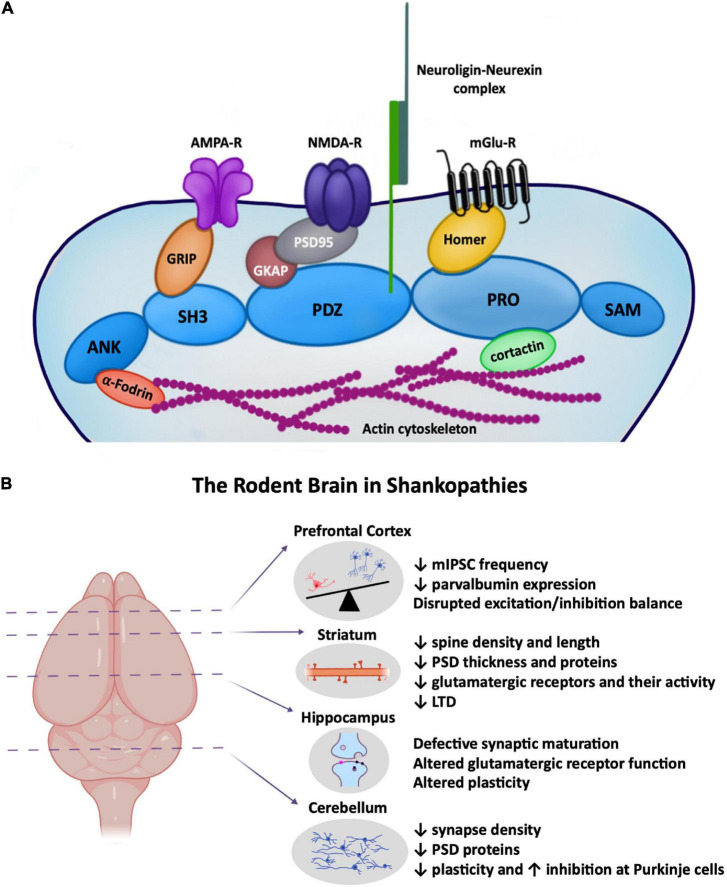
**(A)** Cartoon depiction highlighting the major role of SHANKs in the postsynaptic structure of glutamatergic synapses, linking proteins across the depth of the PSD from the actin cytoskeleton to surface AMPA and NMDA receptors. **(B)** ASD-associated synaptic changes in *Shank* rodent models known to occur in the prefrontal cortex, striatum, hippocampus, and cerebellum. (*Left*) Brain regions highlighted in this minireview where Shankopathies have been most studied. (*Right*) Cartoon depictions of some of the synaptic changes occurring in each region. Created with Biorender.

SHANK2 is among the first proteins to be expressed at the postsynaptic density (PSD), suggesting a key role in synapse development, followed by the recruitment of SHANK3, and then SHANK1 (Boeckers et al., 1999a,b; Grabrucker et al., 2011) which are both implicated in synapse maturation (Sala et al., 2001; Roussignol et al., 2005; Arons et al., 2016). Once localised within the PSD, SHANKs act as major structural and functional scaffolding proteins regulating other PSD proteins, glutamatergic receptors, and proteins in the presynapse through the neuroligin–neurexin trans-synaptic complex (Arons et al., 2012). Predictably ASD-associated mutations in *SHANK* genes have detrimental effects on synaptic function and overall neurological development (Figure 1). Many isoform-specific *SHANK* mutations have been identified in ASD patients (Durand et al., 2007; Berkel et al., 2010; Leblond et al., 2012; Sato et al., 2012), and other neuropsychiatric disorders (for a review see Wan et al., 2021). Here we review the influence of Shankopathies in ASD, in particular how *Shank* mutations impact synapse structure and function in different regions of the rodent brain.

## Hippocampus

The hippocampus plays a critical role in processing multisensory information, memory, cognitive function, and modulating emotion, stress, and social behaviour (Bannerman et al., 2014; Hitti and Siegelbaum, 2014; Zemla and Basu, 2017). Therefore, disruption to the development of hippocampal circuits might underlie social memory impairments and cognitive decline observed in individuals with ASD (Solomon et al., 2016; Cooper et al., 2017; Habib et al., 2019).

SHANK2 is highly expressed in the developing hippocampus, especially during spinogenesis (Boeckers et al., 1999a,b, 2004). Consistently, ASD-associated *Shank2*-specific mouse models show impairments in spatial and working memory, which is dependent on the hippocampus [but also includes other brain regions such as the prefrontal cortex (PFC)] (Schmeisser et al., 2012; Won et al., 2012; Pappas et al., 2017). However, both anatomical and electrophysiological studies have described differential effects of ASD-associated *Shank2* mutations on spine number and morphology, NMDA/AMPA ratio, and synaptic plasticity (Table 1; Schmeisser et al., 2012; Won et al., 2012; Pappas et al., 2017). For example, at Schaffer collateral-CA1 synapses, *Shank2-^Δ*e*6–7–/–^* mice fail to maintain long-term potentiation (LTP) or express long-term depression (LTD) (Won et al., 2012; Lim et al., 2017), whereas *Shank2-^Δ*e*7–/–^* mice display increased LTP with no alterations in LTD (Schmeisser et al., 2012; Wegener et al., 2018). Likewise, mice with mutations in other SHANK homologues showed varying changes in cognition, synaptic structure, function, and plasticity in the hippocampus (Table 1; Hung et al., 2008; Kouser et al., 2013; Wang et al., 2014; Lee E.-J. et al., 2015; Jaramillo et al., 2016). Given the heterogeneity in isoform-specific protein–protein interactions (Boeckers et al., 2005; Shi et al., 2017), distinct spatio-temporal SHANK expression (Boeckers et al., 2004; Grabrucker et al., 2011; Wang et al., 2014), and cell type specific functional outcomes at different synapses (Lim et al., 2017; Kim et al., 2018), the development of differential hippocampal phenotypes in *Shank* mutant animals are not unexpected.

**TABLE 1:** Summary of studies examining shankopathies in the developing brain.

Isoform mutated	Exons and domains targetted	Age and sex	Alterations in comparison to wild-type	ASD phenotype rescued? (yes/no/not attempted)	References
Shank1	Exons 14–15 PDZ domain	3–5 weeks old, males only	Hippocampus: ↓ basal synaptic transmission ↓ mEPSC frequency	Not attempted	Hung et al., 2008
	Exons 14–15 PDZ domain	P25, males only	Somatosensory cortex: ↓ number of PV^+^ neurons, ↓ level of PV, ↓ PV^+^ mRNA levels	Not attempted	Filice et al., 2016
Shank2	Exon 6–7 PDZ domain	3–4 weeks old, male and female	Hippocampus: ↓ NMDA/AMPA ratio ↓ LTP, ↓ LTD	Yes: D-cycloserine (NMDAR partial agonist) and a positive allosteric modulator of mGluR5 normalised NMDAR function and improved social interaction; clioquinol-induced transsynaptic zinc elevation rescues social deficits through postsynaptic Src and NMDAR activation	Won et al., 2012; Lee E.-J. et al., 2015
	Exon 6–7 PDZ domain	P19–23, male and female	Cerebellum: ↓ mEPSC frequency ↓ PSD density ↑ mismatched synapses, ↑ free spine density ↓ GluD2, ↓ GluA1, ↓ GluA2, ↓ GluN2C, ↓ PSD95, ↓ PSD93, ↓ Homer, ↓ VGluT1, ↓ gephyrin	Not attempted	Ha et al., 2016
	Exon 6–7 PDZ domain CaMKII-Cre and Viaat-Cre Shank2*^fl/fl^*	P22–34, females only	Hippocampus (deletion in excitatory neurons): ↓ mEPSC frequency Striatum (deletion in inhibitory neurons): ↓ mIPSC frequency and amplitude ↓ sEPSC frequency	Not attempted	Kim et al., 2018
	Exon 6–7 PDZ domain	P13–28, male and female	Hippocampus: P13–15: ↑ NMDA/AMPA ratio P21–27: ↓ NMDA/AMPA ratio, ↓ NMDA mEPSC, ↓ LTP Medial prefrontal cortex: P13–15: ↑ NMDA/AMPA ratio, ↓ AMPA mEPSC P21–27: ↓ NMDA/AMPA ratio	Yes: chronic suppression of early NMDAR hyperfunction using memantine (P7–21) prevented NMDAR hypofunction and ASD-like social behaviours at later stages (P28–56)	Chung et al., 2019
	Exon 7 PDZ domain	P21–28, male and female	Hippocampus: ↓ mEPSC frequency ↑ NMDA/AMPA ratio ↑ LTP	Not attempted	Schmeisser et al., 2012
	Exon 7 PDZ domain	P25–28, male and female	Hippocampus: ↑ failure rate ↓ NMDA/AMPA ratio, ↑ LTP	Not attempted	Wegener et al., 2018
Shank3	Exons 4–9 ANK repeat domain	3–4 weeks old, males only	Hippocampus: ↓ LTP Cortico-striatal synapses: ↓ NMDA/AMPA ratio	Not attempted	Jaramillo et al., 2016
	Exon 9 ANK repeat domain	P19–54, sex not specified	Hippocampus: ↓ synaptic transmission, ↓ mIPSC frequency Medial prefrontal cortex: ↓ mIPSC frequency	Not attempted	Lee J. et al., 2015
	Exons 13–16 PDZ domain	P6–60, male and female	Striatum (spiny projection neurons): ↑ mEPSC amplitude at P10 ↑ mEPSC frequency at P14 Striatal multi-unit recordings: ↑ AP frequency, ↑ burst frequency, ↑ intra-burst frequency Cortical multi-unit recordings: ↑ AP frequency, ↑ intra-burst frequency	Not attempted	Peixoto et al., 2016
	Exons 13–16 PDZ domain	P15–21, male and female	Striatum (spiny projection neurons): ↑ mEPSC frequency and amplitude	Yes: postnatal downregulation of protein kinase A activity normalised the excessive glutamatergic connectivity in medial striatal neurons, reduced mEPSC amplitude and frequency, and ameliorated the severity of their behavioural phenotypes	Peixoto et al., 2016
	Exons 13–16 PDZ domain	P25, males only	Striatum: ↓ number of PV^+^ neurons, ↓ level of PV, ↓ PV^+^ mRNA levels	Not attempted	Filice et al., 2016
	Exons 13–16 PDZ domain	3 weeks old, male and female	↓ sociability, ↓ social novelty recognition ↑ anxiety	Yes: maternal high zinc supplementation during pregnancy and lactation prevented ASD-deficits in social interaction and anxiety behaviours in juvenile *Shank3^–/–^* offspring, and these effects were maintained into adulthood	Vyas et al., 2020
	Exons 14–16 PDZ domain	P28–35, males only	Striatum: ↓ mEPSC frequency and amplitude	Not attempted	Yoo et al., 2018
	Exon 21 Proline-rich domain	P13–16, males only	Hippocampus: ↓ LTP, ↓ NMDA/AMPA ratio, ↓ mEPSC frequency	Not attempted	Kouser et al., 2013

*LTD, long-term depression; LTP, long-term potentiation; mEPSC, miniature excitatory postsynaptic currents; mIPSC, miniature inhibitory postsynaptic currents; PND, postnatal day; PSD, postsynaptic density; PV, parvalbumin; sEPSC, spontaneous excitatory postsynaptic currents.*

To date, hippocampal synaptic physiology has typically been examined in ASD-associated *Shank* mutant animals post-weaning [> postnatal day 21 (P21)] (Hung et al., 2008; Lee E.-J. et al., 2015; Lim et al., 2017), with fewer studies examining developing hippocampal synapses in pre-weaned animals (Kouser et al., 2013; Jaramillo et al., 2016; Wegener et al., 2018; Chung et al., 2019). *Shank3-^Δ*e*13–16–/–^* mice show enhanced glutamatergic network activity in CA3 pyramidal neurons from birth to the second postnatal week (Chiesa et al., 2019). *Shank2-^Δ*e*6–7–/–^* mice exhibit a developmental switch in NMDAR function at Schaffer collateral-CA1 synapses, from hyperfunction at P14 to hypofunction beyond P21, that was not observed in other *Shank2* mutant mice such as *Shank2-^Δ*e*7–/–^* or *Shank2-^Δ*e*24–/–^* mice (Chung et al., 2019). Interestingly, Schaffer collateral-CA1 synapses in *Shank2-^Δ*e*7–/–^* mice showed reduced AMPAR-mediated synaptic transmission but an enhanced NMDA/AMPA ratio throughout development (Wegener et al., 2018). This suggests that synapse maturation is defective as immature silent synapses, which lack AMPARs but not NMDARs, increase their synaptic strength by incorporating AMPARs (Isaac et al., 1995; Liao et al., 1995; Montgomery et al., 2001). Such disparate developmental alterations in the hippocampus signify that future studies deciphering *Shank*-related synaptopathy need to be conducted in an age-dependent manner.

Multiple treatment strategies have been tested to reverse hippocampal deficits, particularly targetting NMDAR function: D-cycloserine, a partial NMDAR agonist, normalised spatial learning in *Shank2-^Δ*e*6–7–/–^* mice (Won et al., 2012). Moreover, early correction of NMDAR hyperfunction observed during development in *Shank2-^Δ*e*6–7–/–^* mice with the NMDAR antagonist memantine (P7–21) prevented autistic-like social behaviours post-weaning (Chung et al., 2019). Zinc has also been a major focus for reversal of ASD-related synaptic and behavioural deficits (Skalny et al., 2021): acute zinc elevation *via* clioquinol also effectively restored NMDAR function in the hippocampus and prevented spatial memory deficits in *Shank2-^Δ*e*6–7–/–^* mice (Lee E.-J. et al., 2015). Together with the work by Vyas et al. (2020) showing prenatal maternal dietary zinc supplementation can prevent the development of some ASD deficits, these data suggest that amending hippocampal deficits during early brain development may be key to the successful development of effective therapeutic interventions.

## Striatum

The striatum is part of the basal ganglia and in humans consists of the caudate nucleus, putamen, and globus pallidus (Rundles and Papez, 1937). In rodents the dorsolateral striatum (posterior lateral putamen in humans) receives strong projections from sensorimotor cortical regions and has been implicated in mediating the characteristic stereotypic repetitive behaviours in ASD patients (Langen et al., 2009; Lewis and Kim, 2009). SHANK3 is the predominant isoform expressed in the striatum, and mice lacking *Shank3* demonstrate ASD-associated repetitive grooming behaviour (Peça et al., 2011; Wang et al., 2014; Wang et al., 2016; Jaramillo et al., 2016). However, targetted *Shank3* deficiency in the striatum failed to exhibit a repetitive grooming phenotype, although this could be a developmental effect as this phenotype worsens with ageing (Vyas et al., 2020), whereas perseverative exploratory behaviour was observed in mice with *Shank3* disruption in striatal inhibitory neurons (Bey et al., 2018). Examination of striatal synapse structure and function has revealed altered striatal synapse morphologies including decreased spine density and length, and decreased PSD thickness in *Shank3^–/–^* mice. *Shank3* deletion-associated reductions in GluA2, GluN2A, GluN2B, SAPAP, Homer1, and PSD93, and increased mGluR5 expression have also been noted in the striatum (Figure 1 and Table 1; Peça et al., 2011; Mei et al., 2016; Wang et al., 2016). Specifically, complete *Shank3* deletion (*Shank3^Δ*e*4–22–/–^*) causes a significant disruption of the mGluR5-Homer scaffold that decreases mGluR5-mediated signalling but enhances mGluR5 function, highlighting mGluR5 manipulation as a key therapeutic target (Wang et al., 2016).

Similar to what was observed in the hippocampus, different synaptic phenotypes are evident in *Shank* mutant mice. Specifically, during early postnatal development (P13–14), cortical hyperactivity results in increased striatal mEPSC frequency in *Shank3^–/–^* mice (Peixoto et al., 2016). Beyond this developmental time-point, however, the corticostriatal pathway demonstrates reduced field population spike amplitude, NMDAR/AMPAR ratio, NMDAR-mediated EPSCs, mEPSC amplitude and frequency, and impaired LTD (Jaramillo et al., 2016; Wang et al., 2016; Zhou et al., 2016). In comparison to global *Shank3* deletion, GABA neuron-specific *Shank3* deletion showed similar decreases in striatal mEPSC amplitude and frequency suggesting that the suppressed excitatory input onto dorsolateral striatal neurons is induced by the deletion of *Shank3* in striatal GABAergic neurons (Yoo et al., 2018). In addition, targetted disruption of *Shank3* in striatal D1 and D2 neurons resulted in hyper-excitability (Bey et al., 2018), and global *Shank3* loss demonstrated early hyper-excitability which can disrupt the development of corticostriatal circuits (Peixoto et al., 2016). Furthermore, comparisons of the D1 receptor expressing striatonigral neurons (direct projection pathway) and D2 receptor expressing striatopallidal neurons (indirect projection pathway) demonstrated a preferential hypoactivity of the indirect pathway in *Shank3^–/–^* mice (Wang et al., 2017). These structural and functional alterations in striatal synapses, and the imbalance between the direct and indirect pathway of the basal ganglia, are thought to underlie the repetitive grooming behaviour in mouse models of ASD.

Considering the significant deficits caused by the lack of SHANK3 in the striatum (Figure 1), several studies have focused on rescuing neurological and behavioural deficits in *Shank3^–/–^* mice both in adulthood and during development. Adult *Shank3* restoration was found to increase striatal synaptic Homer1b/c, SAPAP3, NR2A, NR2B, and GluA2 levels in *Shank3^–/–^* mice to a level comparable to wild-type mice, as well as significantly increase spine density and mEPSC frequency (Mei et al., 2016). Additionally, *Shank3*-restored mice displayed reduced repetitive grooming and improved social interaction in comparison to *Shank3^–/–^* mice (Mei et al., 2016). Therefore, adult *Shank3* restoration improves striatal neurotransmission and rescues ASD-associated behavioural deficits in *Shank3^–/–^* mice, showing that some of the developmental neural deficits caused by lack of *Shank3* can be reversed. However, complete rescue of ASD behaviours was not achieved, with anxiety and motor coordination deficits remaining after *Shank3* restoration. Recent work has examined whether therapeutic strategies targetted during brain development can also influence ASD-associated behaviours and striatal synaptic physiology: supplementation of dietary zinc, which is known to ameliorate ASD-associated behaviours post-weaning (Fourie et al., 2018), is also effective during brain development in *Shank3^–/–^* pups when given to female mice during pregnancy and lactation. Maternal dietary zinc supplementation regulated NMDAR-mediated glutamatergic function, rescued pre-synaptic deficits, and prevented LTP in the cortico-striatal pathway in *Shank3^–/–^* offspring mice. ASD-associated repetitive grooming behaviours were also rescued in these mice (Vyas et al., 2020). Furthermore, postnatal downregulation of protein kinase A (PKA) activity normalised the excessive glutamatergic connectivity in medial striatal neurons in *Shank3^–/–^* mice and ameliorated the severity of their behavioural phenotypes (Peixoto et al., 2016). These studies exhibit that *Shank3*-deletion associated striatal deficits have the potential to be prevented when strategies are applied during brain development.

## Sensory Cortex

For each of our senses there are dedicated parts of the cortex that process each type of information (visual, auditory, somatosensory, olfactory, and gustatory). In ASD, sensory disturbances present as a complex set of symptoms ranging from hypersensitivity to hyposensitivity. As the sensory cortex is readily accessible for *in vivo* recording, several studies have examined sensory processing in *Shank* models of ASD. Multiunit recordings in adult *Shank3* heterozygote rats revealed that auditory responses in the primary auditory cortex and secondary auditory areas were reduced (Engineer et al., 2018). Similarly, auditory responses to speech sounds were weaker in the primary auditory cortex (Engineer et al., 2018). Interestingly, these responses could be enhanced through reward-based speech sound discrimination training, suggesting that these deficits can be corrected with speech therapy. In contrast, adult *Shank3B^–/–^* mice showed normal auditory processing and enhanced pitch discrimination (Rendall et al., 2019), and postnatal *Shank^+/–^* mice showed normal startle responses to loud sounds (Orefice et al., 2016), suggesting that auditory processing is largely normal in *Shank* mutant mice both during development and in adulthood. When tactile stimulation was included, however, startle responses were enhanced in postnatal *Shank* mutant mice (Orefice et al., 2016). In this tactile prepulse inhibition test, where a tactile stimulus in the form of an airpuff directed at the skin precedes an auditory stimulus, postnatal ASD mutant mice showed increased responses. Furthermore, the ASD mice showed enhanced startle responses to the airpuff alone, and an inability to discriminate in a textured version of novel object recognition. This suggests hypersensitivity to touch plays an important role in ASD sensory behaviours from early development.

Somatosensory hypersensitivity has also been observed in the barrel cortex in response to whisker stimulation in adult *Shank3B^–/–^* mice (Chen et al., 2020). Using *in vivo* calcium imaging in excitatory and inhibitory layer 2/3 neurons this was found to be due to reduced responses in GABAergic neurons, which in turn increased responses in cortical pyramidal neurons. Interestingly, lack of *Shank3B* specifically in GABAergic neurons in the somatosensory cortex leads to increased pyramidal responses and hypersensitivity to whisker stimulation. This study reveals that SHANK3 in GABAergic interneurons functions to strengthen the excitatory input to these neurons, increasing inhibitory network participation and balancing the network. Without SHANK3 in interneurons, they respond less to tactile stimuli and excitation dominates, leading to hypersensitivity (Chen et al., 2020), demonstrating the importance of correctly functioning SHANK3-containing synapses for balancing excitation/inhibition for normal sensory responses and behaviour. Furthermore, *Shank3B^–/–^* mice show reductions in the number, intensity, and size of parvalbumin-positive terminals on pyramidal neurons in cortex (Gogolla et al., 2014).

During early development, the somatosensory cortex of *Shank3B^–/–^* mice is hyperactive (Peixoto et al., 2016). This heightened cortical activity feeds forward into the striatum, resulting in increased corticostriatal connectivity in *Shank3B^–/–^* mice. This shows that cortical hyperactivity can have feedforward effects that contribute to ASD behavioural phenotypes that aren’t typically considered to be sensory, such as increased grooming.

## Prefrontal Cortex

The PFC interconnects subcortical areas including the nucleus accumbens, limbic cortex, ventral tegmental area (VTA), amygdala, and hypothalamus, underpinning cognitive processes, regulation of emotions, sociability, and motivation (Miller and Cohen, 2001). The medial prefrontal cortex (mPFC) is specifically involved in higher cognitive function and social behaviour, and is implicated in many neurological disorders such as ASD and schizophrenia (Alexander and Brown, 2011).

SHANK1, 2, and 3 are highly expressed in the PFC (Monteiro and Feng, 2017). *Shank1* and *Shank3* deficient mice exhibit reduced expression of parvalbumin in the mPFC early in development (Filice et al., 2016), and a reduced number of parvalbumin interneurons is also observed in postmortem tissue from ASD patients (Hashemi et al., 2017). The frequency of spontaneous inhibitory synaptic activity is decreased in the mPFC in young *Shank3^Δ*e*9–/–^* mice (P19–25), resulting in enhanced excitatory activity (Lee J. et al., 2015). The disruption in the balance between neuronal excitation and inhibition in the mPFC has been elucidated as one of the major cellular mechanisms contributing to social deficits in ASD model animals (Figure 1; Xu et al., 2019), and indeed social interaction deficits are prevalent in *Shank* ASD mice (Table 1). Moreover, increasing the excitability of parvalbumin interneurons in the mPFC has been shown to rescue social deficits (Selimbeyoglu et al., 2017; Cao et al., 2018).

Several mechanistic studies have now demonstrated downstream elements involved in the improvement of social deficits and/or synaptic dysfunction in the mPFC caused by *Shank* deficiency in young mice. This includes restoration of F-actin by inhibiting cofilin, resulting in reversal of NMDAR hypofunction and sociability deficits (Duffney et al., 2015). Other targets include histone deacetylase 2 (HDAC2) transcription, and the *Shank3* gene itself, with HDAC inhibitors or re-expression of *Shank3* alleviating social deficits (Qin et al., 2018; Lee et al., 2021). Restoration of *Shank3* in the anterior cingulate cortex, which is the association cortices like PFC and its role is emphasised in sociability, also improved social deficits (Guo et al., 2019). Targetting pathways into the mPFC from the hippocampus with oxytocin treatment, which is required for the formation of social recognition memory, also attenuates the changes in synaptic plasticity as well as social behaviour deficits during development (Harony-Nicolas et al., 2017). Using designer receptors exclusively activated by designer drugs (DREADDs), chemogenic activation of pyramidal neurons in the mPFC can rescue NMDAR hypofunction and social deficits in a *Shank3* haploinsufficiency model (Qin et al., 2019). These findings together identify future therapeutic strategies for ASD specifically in relation to PFC dysfunction and social deficits.

## Other Brain Regions

While the majority of *Shank*-related ASD research has focussed on the hippocampus, cortex, and striatum, it is clear that other brain regions are also affected, reflecting the heterogeneous nature of ASD. The VTA modulates cortical and striatal networks and as such contributes to social deficits in ASD. Downregulation of SHANK3 specifically in the VTA during early development slows excitatory synapse maturation, reducing the excitability of dopaminergic neurons and altering excitation/inhibition balance (Bariselli et al., 2016). Potentiation of mGluR1 activity during the developmental period rescues dopaminergic neuron function and prevents social deficits into adulthood, and this effect can be mimicked by direct optogenetic activation of these neurons, highlighting the importance of VTA dopaminergic neuron activity in social behavioural deficits in ASD.

Other lesser studied brain regions affected by ASD-associated mutations in *Shanks* include the cerebellum and hypothalamus. The cerebellum is a site of pathology in ASD aetiology, with behavioural deficits in motor learning, motor coordination, and social interaction dependent on cerebellar changes. Lack of *Shank2* reduces excitatory synapse density, levels of postsynaptic proteins, and reduced plasticity at Purkinje cell synapses (Figure 1; Ha et al., 2016; Peter et al., 2016). Excitation/inhibitory balance is also altered – with an increase in inhibitory input onto Purkinje cells observed. Restricting *Shank2* deletion to the cerebellum results in enhanced repetitive and anxiety behaviours (Ha et al., 2016), linking ASD-associated mutations in *Shank2* in the cerebellum with specific ASD behaviours. In human patients, brain imaging studies have revealed cerebellar vermis hypoplasia with Phelan–McDermid Syndrome, a neurodevelopmental disorder with ASD-like features that is associated with the loss of one copy of *SHANK3* (22q13.3 deletion). However, this was not observed in all 22q13.3 patients, suggesting SHANK3 disruption is not causative for abnormal development of posterior fossa structures (Aldinger et al., 2013).

The hypothalamus is another brain region implicated in the development of ASD, especially in social behaviour and pup rearing deficits. Indeed lack of *Shank2* results in lack of attachment and decreases social bonding between mother and pups as evidenced by offspring neglect (Grabrucker et al., 2021). Low levels of neuronal activation were observed in the medial preoptic region of the hypothalamus and the associated regions of these mice. Increasing activity in this region using DREADDs re-established social bonding in ASD mice, providing strong anatomical and physiological information on the biological origin of the development of this ASD deficit. Altered neurotransmitter systems are evident early during postnatal development in the hypothalamus, with neonatal mice lacking *Shank3* showing decreases in GABAergic and glutamatergic markers, but increases in cholinergic, dopaminergic, and serotonergic markers that may underpin adult ASD phenotypes (Bukatova et al., 2021).

Beyond synaptic effects, electrophysiological studies in multiple brain regions have shown ASD-related *Shank* mutations also influence hyperpolarization-activated cyclic nucleotide-gated channel proteins (HCN proteins) that form Ih channels (Yi et al., 2016; Zhu et al., 2018). In the ventrobasal (VB) complex of the thalamus, VB thalamocortical relay neurons highly express HCN channels which enable switching between tonic and burst firing modes. These neurons also highly express SHANK3, and ASD-related SHANK3 mutations reduce Ih current and alter action potential firing (Zhu et al., 2018). SHANK3 mutations expressed in embryonic-stem cell derived human neurons also have been shown to specifically impair Ih channels (Yi et al., 2016). These SHANK-related inductions of Ih channelopathy influence essential neuronal properties such as resting membrane potential and action potential firing and show an important function of SHANKs in regulating electrical properties outside of the synapse that may underpin ASD-related cellular mechanisms.

The expression of SHANKs in the peripheral nervous system (PNS; Raab et al., 2010) raises the idea that some ASD phenotypes will include peripheral synaptic pathology, however, this is an understudied area. Shank2/3 expression has been described at the neuromuscular junction as well as at synapses of the superior cervical and myenteric ganglion (Raab et al., 2010). Growing evidence suggests that ASD-genetic mutations also alter peripheral mechanisms that contribute to ASD-associated sensory abnormalities including tactile hypersensitivity (Orefice et al., 2019; Schaffler et al., 2019). Peripheral GABA_*A*_ receptor agonist treatment can reduce tactile over-reactivity, as well as improve anxiety behaviours, body condition, and some social impairments (Orefice et al., 2019). Interestingly, memory and motor impairments, and repetitive behaviours were not altered, strengthening the evidence that the PNS plays a strong role in a subset of ASD-associated behaviours.

## Future Challenges

Multiple brain regions are clearly significantly affected in ASD from the synaptic to the network level (Figure 1). The resulting effects on neural activity potentially result in miswiring of the developing brain, altering both output and communication between these regions to collectively elicit the complex social behavioural deficits in ASD (Fourie et al., 2018; Han et al., 2020). Significant challenges exist for the development of therapeutics in ASD, including (1) the differential effects of ASD on neuronal structure and function across brain regions, even within specific genetic variants, (2) the differential effects of varying ASD-associated mutations, (3) fewer cellular studies examining ASD effects during early brain development, and (4) the different effects observed in neuronal function across developmental stages when these have been examined. Added to this is the often-unrecognised limitation that many studies perform functional experiments during the postnatal development but compare these to structural changes occurring in adult tissue. Together these temporal, functional, and location-specific differences will likely necessitate multiple therapeutic options that are specifically targetted to counterbalance differences in *Shank* mutation, affected brain region, and developmental time points.

## Author Contributions

YV, JC, KL, YJ, and JM developed the writing plan and drafted the manuscript. YV and PC developed the figure. All authors approved the final manuscript.

## Conflict of Interest

The authors declare that the research was conducted in the absence of any commercial or financial relationships that could be construed as a potential conflict of interest.

## Publisher’s Note

All claims expressed in this article are solely those of the authors and do not necessarily represent those of their affiliated organizations, or those of the publisher, the editors and the reviewers. Any product that may be evaluated in this article, or claim that may be made by its manufacturer, is not guaranteed or endorsed by the publisher.
